# Exploring the Role of Sickness Absence Coordinators in Implementing Interventions to Reduce Sickness Absence in Public Sector Workplaces in Denmark

**DOI:** 10.1007/s10926-024-10183-1

**Published:** 2024-03-22

**Authors:** Lene Rasmussen, Maj Britt Dahl Nielsen, Anne Helene Garde, Jesper Kristiansen

**Affiliations:** 1 The National Research Centre for the Working Environment, Lersø Parkallé 105, 2100 Copenhagen, Denmark; 2 The National Institute of Public Health, University of Southern Denmark, Studiestræde 6, 1455 Copenhagen, Denmark

**Keywords:** Absenteeism, Return to work, Rehabilitation, Workplace, Implementation, Intervention

## Abstract

**Purpose:**

In 2019, an initiative to reduce sickness absence in public sector workplaces in Denmark was introduced. The initiative involved appointing a sickness absence coordinator (SA coordinator) to oversee the implementation of workplace-based sickness absence interventions. Since the role of the SA coordinator is a novel concept introduced as part of the initiative, this study investigates the responsibilities of SA coordinators and the challenges they experienced in fulfilling this role during the implementation process.

**Methods:**

Semi-structured interviews with and observations of SA coordinators from four public sector workplaces were carried out. We collected the first four interviews and observations during the implementation process with follow-up interviews collected at the end of the process. The data were analyzed using thematic analysis.

**Results:**

The SA coordinators all experienced challenges in terms of lack of commitment among the line managers to participate in the intervention. They experienced being seen as a burden rather than a helping hand, and felt that the line managers might have difficulty recognizing the value of the SA coordinators. Potential ways to improve the collaboration between the SA coordinators and the line managers include considering hiring the SA coordinator in-house and incorporation of intervention components into existing activities to accommodate the busyness of the line managers.

**Conclusions:**

To support the SA coordinators in carrying out their role and responsibilities, this study suggests that it is important to ensure commitment to the role, especially among the line managers in order to enhance good working relationships.

## Background

Designating an in-house person to facilitate the return to work (RTW) process for sick-listed employees is common practice in many workplaces, as the RTW process can be complex and difficult for different stakeholders to navigate within [1, 2]. In the scientific literature, RTW coordinators are defined as “individuals employed to plan and facilitate return to work of workers who are absent from work due to injury or illness” [3] with the main goal of increasing RTW after work disability [4]. Work responsibilities include providing individual support to and contacting employees on sick leave, collaborating with different stakeholders involved in the RTW process [5, 6], facilitating work accommodation, developing and implementing individually tailored RTW plans [7, 8], identifying barriers for RTW, and applying laws, policies and regulations related to sickness absence and RTW [4, 6, 9]. Research has demonstrated that RTW coordinators play an essential role in the success of RTW programs [8, 9] and that workplace interventions including RTW coordinators may result in shorter durations of sickness absence [10].

In Denmark, a similar concept to RTW coordinators was developed in connection with the *Sickness Absence Initiatives* from 2019, which is an initiative from the previous government that aims to reduce sickness absence and increase work participation in the public sector. The initiative included the implementation of sickness absence interventions at public sector workplaces with high levels of sickness absence, facilitated by a designated sickness absence coordinator (SA coordinator). In the description of the initiative, there are no strict requirements for the role of the SA coordinators, as it is up to the workplaces themselves to decide the content and work responsibilities of the role. This means that the role can differ from workplace to workplace. However, it is stated in the description that the SA coordinators are the key drivers of the interventions, with the responsibility of ensuring competences, resources and focus to reduce sickness absence. Their main tasks are to provide counselling to line managers about the management of sickness absence and to ensure the implementation of intervention activities through coordination and communication [11].

Largely, SA coordinators and RTW coordinators are similar concepts, as both roles are responsible for facilitating RTW processes for employees on (long-term) sick leave. However, where RTW coordinators primarily take action when an employee is already on sick leave and are more in direct contact with the employee, the SA coordinators in the present study are attributed a proactive role also focusing on preventing sickness absence from occurring in the first place. Also, their role is primarily about supporting line managers in dealing with sickness absence, rather than supporting the employees directly [3, 4, 11–13]. The role and experiences of RTW coordinators have been widely examined. For example, in a recent study by Hopwood et al. exploring RTW coordinators perceptions of their role, it was found that the participating RTW coordinators saw themselves as “trust builders, experts, detectives and motivators” [14] and highlighted their experiences with supporting sick-listed employees. When it comes to sickness absence, previous research argue that line managers play a key role in preventing and managing sickness absence [15, 16]. However, line managers also very often experience challenges in this role, for example, because they lack training or organizational support [17, 18]. Since the SA coordinators play a central part in the implementation of the sickness absence interventions with a particular focus on supporting the line managers, it is relevant to generate knowledge about the role and the challenges the SA coordinators experience. In practice, this knowledge can potentially contribute to reduced absenteeism, which could otherwise have consequences for both the individual, the workplace, and society [19].

On this background, we intent to explore the following research question: What is the role of SA coordinators and which challenges did they experience during the implementation of workplace-based sickness absence interventions?

## Methods

### Study Design

The study is a qualitative study combing two qualitative methods (semi-structured interviews and non-participant observations). Data comprises multiple individual interviews with SA coordinators as well as observations of their work in different public sector workplaces in Denmark. Reporting of the study will follow the Consolidated Criteria for Reporting Qualitative Research (COREQ) [20].

### Context

In Denmark, the RTW process of sick-listed employees is managed by the workplaces in cases of short-term sickness absence (typically < 30 days), whereas when the sickness absence is longer it is managed by the municipalities where case managers develop plans and goals for the process [21]. Absent employees are eligible to receive sickness benefits for up to two years from the first day of absence [21]. The financial expenses lie with the employer for the first 30 days, after which the responsibility transfers to the municipalities [22]. This means that employers have limited financial consequences as a result of long-term sickness absence. However, workplaces still feel the direct and indirect consequences of sickness absence, and are therefore central arenas for preventing and reducing sickness absence. Previous research has shown that workplace-based efforts can have a positive effect on sickness absence [23]. The foundation of this study is an evaluation of the *Sickness absence initiative*. One component of the initiative was the establishment of a fund from which public sector workplaces could apply for funding for local workplace-based interventions to reduce sickness absence. A comprehensive framework for the interventions was developed based on best-practices and informed by scientific evidence [11]. The framework is described in detail elsewhere [24], but in brief it consists of five components that the workplaces are obliged to implement: (1) clear and standardized procedures on how to manage sickness absence; (2) initiatives to strengthen the psychosocial or physical work environment; (3) data on sickness absence and well-being in the organization that is actively used in the prevention and management of sickness absence; (4) establishment of a decision-making forum with broad representation from management and employees to ensure that the intervention is deeply rooted in the organization; (5) a coordinating body that ensures coherence and coordination of all sickness absence activities in the organization (the SA coordinators) [11].

The workplaces, which received funding (a total of 43 workplaces), had to implement the framework over a two-year project period and it was necessary to implement all five components since it is assumed to be the combination of the components, with the SA coordinator as the driving force, that leads to a reduction of sickness absence [11].

### Participants

The SA coordinators participating in this study were recruited from four public sector workplaces in Denmark, which were among the 43 workplaces that received funding to implement local sickness absence interventions. Recruitment took place in the fall of 2020. In order to examine the implementation process in different contexts, we used purposeful sampling [25] to recruit workplaces from all levels in the Danish public sector (governmental, regional and municipal). We contacted the project managers at each of the four workplaces, who all agreed to let us examine the implementation of their sickness absence intervention. The workplaces differed in size (120–4500 employees) and represented health care, day care and the judicial system. The designated SA coordinators from each of the workplaces where selected a priori to participate in interviews and observations due to their central role in the interventions. We contacted them per e-mail to arrange a meeting about their participation. All four participants agreed to participate and verbal informed consent was obtained prior to the interviews. For three of the workplaces, the interviews were done with the same participant, with changes in participants in the fourth workplace due to staff replacements. Due to the low number of participants, ensuring their anonymity was of utmost priority. Therefore, personally identifiable information such as names, locations, and events has been removed from the transcripts and the quotes used in the "Results" Section. For the same reason, descriptions of the workplaces are omitted and we only refer to the participants as SA coordinator 1–4.

All the recruited SA coordinators had a professional background within HR. SA coordinator 1 (which consisted of a team from which one representative was chosen to participate in the data collection) was permanently employed in the local HR department and had other responsibilities than facilitating the intervention. SA coordinators 2–4 worked on a fixed-term contract ending their employment after the implementation process. These SA coordinators were hired specifically to facilitate and coordinate the implementation of the interventions.

### Data Collection

A total of eight interviews and 24 observations (e.g. of meetings, workshops) were collected for this study. Each SA coordinator was interviewed twice with the first interviews held in May and June 2021 (during the implementation process) and follow-up interviews in June, July and August 2022. The first interviews gave us unique insight into the role of the SA coordinators and their experiences while they were working on implementing the interventions. The follow-up interviews gave us the opportunity to view the implementation process retrospectively. At the same time, the follow-up interviews provided more in-depth reflections as the SA coordinators had more time to reflect upon their role. The combination of the two interview rounds therefore gave us a comprehensive insight into the role and responsibilities of the SA coordinators and their experiences during the implementation process, both in real time and in retrospect.

For the first interviews, we developed an interview guide containing questions about the role of the SA coordination. Specifically, we asked them to describe their role as SA coordinators, including work tasks and how they collaborated with others. We also asked them which attitudes they met from others in the process and how they would evaluate their role. Examples of questions are “Can you describe your role as SA coordinator?”; “Can you describe how you collaborate with the line managers?”; and “Regarding your role, what do you think works well and what works less well?”

We developed another interview guide for the follow-up interviews, focusing on their overall experiences with implementing the interventions, which challenges they met in the process, learnings and the forward-looking process. Examples of questions are “Are there any parts of your role that you think you managed to do well? Or less well?”; and “Is there anything in the role as SA coordinator that you would change? For example, work tasks and responsibilities?”

All interviews were carried out by the first author (LR) and had a duration between 55 min and 1,5 h. The interviews were held online due to the COVID-19 pandemic except for one interview, which was held in person in connection with the observations. All follow-up interviews were carried out online. All interviews were recorded and transcribed verbatim in NVivo 12.

The 24 observations were divided between the SA coordinators, with 7 observations of SA coordinators 1 and 2, respectively, 4 observations of SA coordinator 3 and 6 observations of SA coordinator 4. Observations were carried out in June 2021, October 2021 and March 2022, which was during the implementation period. The purpose was to observe the role of the SA coordinators in practice to gain an understanding of the work responsibilities of the role. The observations are used as a supplement to the collected interview data. We developed an observation scheme with two main foci: (1) describing the SA coordination, including the role, competencies and organizational anchoring and (2) examining what SA coordinators do to fulfill the coordinating and facilitating role that the intervention framework attributes to them (i.e. which activities they initiate and with whom they communicate and collaborate and how, etc.). LR did all the observations and wrote field notes.

### Data Analysis

Both the interviews and the field notes were analyzed using inductive thematic analysis to identify relevant themes in the data [26]. The themes were generated by reading and re-reading the transcripts to identify reoccurring patterns across the data. These patterns laid the foundation for the initial codes, which were tested on two interviews before being revised. Coding was done in NVivo 12. The initial codes were compared to each other and organized into subthemes capturing the commonality among the codes. These subthemes were then compared and categorized under four main themes that capture the similarities of the subthemes. This process was carried out by the first author (LR), who, with input from the co-authors, further redefined and sharpened the themes.

## Results

**Fig. 1:**
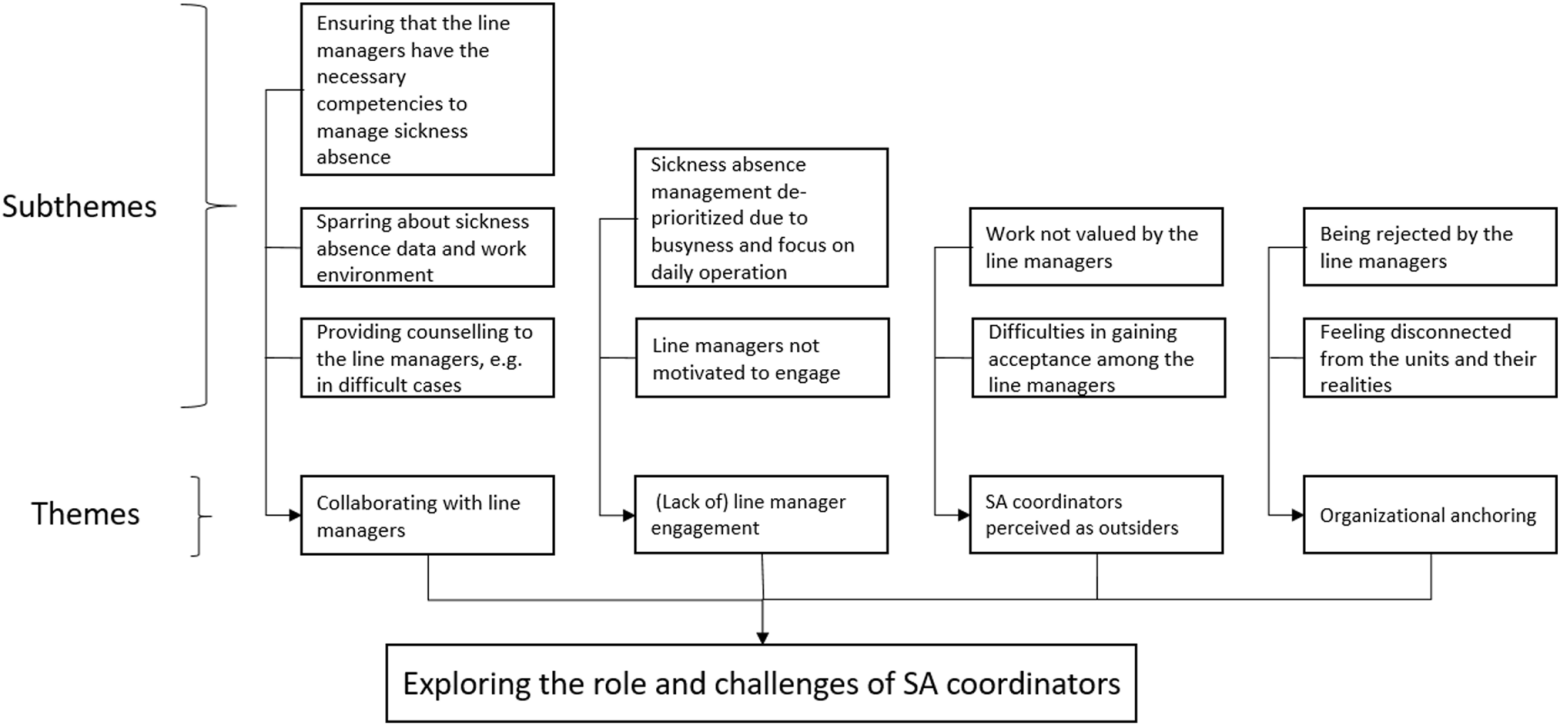
Themes and subthemes identified in the data

The thematic analysis resulted in four themes and nine subthemes (illustrated in Fig. 1). The four themes will form the structure in the "Results" Section.

Although the workplaces, as described, had free rein to determine the specific tasks and role of the SA coordinator, it became evident during the analysis that the participating SA coordinators largely shared similar work duties. Hence, there is not significant variation in how the role is designed across the respective workplaces. During the analysis, it became clear that one of the main responsibilities of the SA coordinators was to support the line managers in managing sickness absence, which is why the findings presented here focus on how the SA coordinators support the line managers and their experiences with this. In this study, these line managers are frontline managers from day care, health care and the judicial system, responsible for both daily operations and personnel management.

### Collaborating with Line Managers in Managing Sickness Absence

According to the SA coordinators, the collaboration with the line managers includes providing counselling to the line managers about sickness absence among their employees. The SA coordinators mention supporting the line managers in difficult cases, for example, in case of dismissal due to excessive absences, or if there are other factors that hinder the line managers in managing the absence appropriately. An example of this was observed during a meeting between one of the SA coordinators and a line manager where the line manager asked for help with a case due to her close relationship with the employee in question and needed advice on how to handle it.

The SA coordinators also mention that they spar with the line managers about data on sickness absence focusing on developments, patterns and whether the line manager should pay particular attention to certain employees at risk of sickness absence. It also includes sharing knowledge on work environment such as which work-related factors that may cause sickness absence and which preventive effort that can be done to prevent it from occurring. Finally, the SA coordinators also make sure that line managers possess the required skills to handle sickness absence, as it is the responsibility of line managers to practically manage sickness absence. Nevertheless, our analysis of the data revealed various challenges faced by the SA coordinators, which likely influenced the intended close collaboration. These challenges are outlined below.

### Lack of Line Manager Engagement

Due to the SA coordinators’ intended close collaboration with the line managers, the SA coordinators highlight the importance of ensuring engagement from the line managers to actively participate and support the sickness absence interventions that are implemented in the work units. However, all the SA coordinators have experienced lack of engagement from the line managers during the implementation process. One example is that the interventions, which the SA coordinators are responsible for, are initiated by the top management and not by the line managers. Although the SA coordinators express that there is consensus in the work units that it is necessary to reduce sickness absence, the SA coordinators may experience resistance from the line managers, as they have not been involved in decisions related to the interventions, for example which intervention activities to implement:

”It can be challenging because [the line managers] didn’t ask for this intervention. So it must be difficult for them to suddenly allocate resources and focus on a project that they have not been involved in from the beginning and just be told: You have to because we need to reduce our sickness absence.” (SA coordinator 2).

Not being involved could lead to the line managers not being motivated to support the intervention or at least it will be more difficult to get them onboard. This was also observed during the observations where a SA coordinator told us about experiencing resistance from a line manager who was not included in the decision to initiate the intervention and was therefore not motivated to support the activities.

Another aspect of the collaboration between the SA coordinators and line managers is that some line managers, according to the SA coordinators, want to see results (i.e. reduced sickness absence) but do not want to make an effort. However, the SA coordinators say that they need the participation from the line managers, as the effort to reduce sickness absence is a shared responsibility. One SA coordinator elaborates:

“I was hired to solve something that I can’t do myself. It’s sometimes difficult to get them to understand that I need their commitment.” (SA coordinator 3).

According to the SA coordinators, the lack of commitment does not necessarily mean that the line managers do not think that high levels of sickness absence is problematic but it is difficult to allocate time for the activities in a busy day. Busyness among line managers is another challenge that the SA coordinators experience having to navigate within and it can be difficult to get the line managers to prioritize sickness absence management because daily operation is their main priority. The work units in this study come from work areas that are challenged by staff shortages, meaning that the line managers focus on making sure that operations are running despite lack of staff.

According to one of the SA coordinators, commitment from the top management would be a way of enhancing engagement and participation, as the commitment from the top management is reflected in the rest of the organization.

”It’s also a task for the top management level. Because it has a signal value that the top management says: This is important and we must work with it. And then it’s not up for discussion.” (SA coordinator 4).

The quote indicates that when the top management acknowledges the importance of making efforts to reduce sickness absence, this acknowledgement will likely be present among the line managers as well, which can result in increased motivation to actively engage in the intervention.

### SA Coordinators Perceived as Outsiders

Most of the SA coordinators have been hired specifically for the interventions. Coming from the outside can pose special challenges. Some of the SA coordinators experience that the line managers tend to perceive them as external consultants, who interferes with their usual approaches, which can make it difficult to gain acceptance from the line managers. Some of the SA coordinators find that, due to staff shortages, the attitude among some line managers is that it would be more beneficial to hire more staff for operations (such as nurses and health care workers) instead of a consultant. In relation to this, the SA coordinators mention that line managers with high seniority feel that they have everything under control and that they, due to their years of experience, knows better than the SA coordinator and therefore do not need their help.

”[…] And then trying to build relations. I mean, if someone from the outside suddenly tells you what to do, then you tend to think: Relax, I’ve been here longer than you! So in that way, it can be a challenge to be a coordinator if you are hired from outside the organization.” (SA coordinator 2).

### Organizational Anchoring

All of the SA coordinators are organizationally anchored in HR. The benefit of this is that they often have other resources to draw on (such as work environment consultants, HR and legal competences, e.g. to ensure appropriate dismissals), which the SA coordinators find helpful as it supports them in providing adequate support to the line managers. However, the downside of being anchored within HR, and often physically placed outside of the work units is, to some of the SA coordinators, that they feel distanced from the work units. The SA coordinators say that it can be challenging to understand the reality of the work units, i.e. understand the local realities and what the line managers need from the SA coordinators to help them manage and reduce sickness absence:

*“*We tend to be a bit disconnected from what is going on in reality, that is in our work units, which has been a challenge, I would say” (representative from SA coordination 1).

It is possible to imagine that not being able to fully understand the reality of the work units causes difficulties in providing the necessary support, as it is not clear which help is needed in the work units. It can also mean the SA coordinators and the line managers may have different perceptions of what must be done in the work units to help reduce sickness absence. This discrepancy may also affect the collaboration between the SA coordinators and the line managers, as they may not work towards the same goal.

Previously, we presented how the SA coordinators experienced being perceived as outsiders. This, together with their physical distance from the work units, can mean that the line managers do not perceive them as a natural part of the organization and therefore they may be less inclined to let the SA coordinators into their work units, and thus also allocate time and resources, to carry out relevant initiatives.

## Discussion

This study examined the roles of SA coordinators from four public sector workplaces in Denmark and the challenges they experienced during the implementation of workplace-based sickness absence interventions. In the introduction, we argue that the roles of the SA coordinator and RTW coordinators differ in that SA coordinators are intended to have a proactive focus on sickness absence and support line managers in preventing and managing sickness absence, while RTW coordinators focus on supporting the RTW process of sick-listed employees directly. This argument found support in the results, as it is clear that one of the main responsibilities of the SA coordinators is collaborating with and supporting the line managers in managing sickness absence, including to spar with the line managers about sickness absence data and work-related factors behind sickness absence. However, the SA coordinators interviewed for this study highlighted a number of challenges they encountered in this collaboration, most of which were related to challenges in being perceived as a valuable help by the line managers. These challenges made it difficult for the SA coordinators to build trustful working relationships with the line managers and could potentially be seen as barriers for collaboration.

The concept of interprofessional collaboration refers to professionals from different disciplines working together to achieve a common goal. It has often been investigated in health care settings, such as presented in a review by Schot and colleagues [27] and a paper by Engel and Dawn [28], focusing on health care professionals working together to provide services for health care users and improve patient outcomes. As this study shows, interprofessional collaboration occurs in other settings and other constellations. In a literature review, D’Amour et al. [29] found that interprofessional collaboration, among other things, must include mutual trust and respect, interdependency and awareness of and valuing each other’s perspectives and contributions. This is similar to a qualitative study exploring how interprofessional collaboration can be improved by Wei et al. [30] where they argue that building caring relationships with trust and respect between stakeholders is crucial for collaboration. Our findings indicate that these factors can be difficult to achieve. For example, the SA coordinators feel that the line managers and SA coordinators focus on different goals (ensuring efficient operation versus reducing sickness absence). Although it must be anticipated that a reduction in sickness absence will lead to better daily operations, the SA coordinators express that the line managers tend to be more focused on acutely securing enough staff, for example by calling in substitutes or changing work schedules to make ends meet.

As for the mutual trust and respect and awareness of and valuing each other’s perspectives and contributions, we found that some of the SA coordinators experienced that the line managers saw them as an external consultant from outside the organization, telling them what to do. It may feel provocative for the line managers when a newly hired person wants to interfere with the way things are done. It may be because the line managers, according to the SA coordinators, are of the opinion that they themselves know their employees best and know what works. In addition, the SA coordinators try to get the line managers to initiate activities the line managers do not feel they have time for. Furthermore, some of the SA coordinators were met with an opinion that it would make more sense to hire more staff in the daily operation instead of a consultant. The SA coordinators can therefore be considered an extra burden for the line managers rather than a support function. Having said that, the results do not directly indicate mistrust or disrespect, but it does indicate that some of the line managers, according to the SA coordinators themselves, do not always value their role.

How can collaboration then be improved? First of all, inspired by the paper by D’Amour and colleagues [29], it is arguable that setting a common goal that both the SA coordinators and the line managers can agree to, and that creating an understanding that they are dependent on each other’s competences and knowledge in order to succeed in reducing sickness absence and thereby also secure efficient daily operation, could improve their collaboration. In the review by Schot et al., focusing on different goals is referred to as a gap that “exists between professional perspectives” [27], which can be overcome by focusing on what they have in common. In another study by Martin et al., this is described as ‘*communal stories* that help diverse stakeholder groups to develop a sense of what they have in common with each other’ [31]. Translated to this study, collaboration may be enhanced by creating a shared narrative around a desire to retain employees in the workplace.

Second, the findings suggest that it would be more advantageous to consider designating the role of SA coordinator to a person internally within the organization, someone who is already known in the organization and knows the relevant policies and tools. In this way, there is a higher chance that the line managers already have some form of working relationship with the SA coordinator, which makes it easier to build trust and create acceptance of the role. Mellor et al. [32] argue that using in-house competences to facilitate interventions is helpful as these already have contextual knowledge and insight into the organization’s needs. Another advantage of using in-house competences is that these competences will remain in the organization and can help sustain changes after the implementation process, whereas most of the SA coordinators in this study are employed on a fixed-term contract, ending their employment after the project period.

Third, the findings suggests that it is important to ensure commitment from the top management to the sickness absence intervention, as this commitment is reflected in the rest of the organization. This organizational commitment will enhance the SA coordinator’s possibilities to work closely in connection with the workplaces, which previous research has recommended [33]. Research has found commitment from the top management to be important contextual factor in sickness absence interventions [34, 35]. However, the role of line managers in implementing interventions has been highlighted in previous research as well [32, 36, 37]. Line managers are described as the drivers of change [35, 38], underlining the need for them to actively engage in the intervention as they influence both the implementation and the outcome of the intervention. This was also observed in our data. Nielsen [39] argue that decisions on initiating interventions often lies with the top management but the execution of the intervention activities is distributed to the line managers. It is argued that if the line managers do not feel ownership or feel like they have no saying in the implementation process, they are less likely to support the intervention.

Additionally, line managers have to navigate a busy workday with a focus on ensuring efficient daily operations, which means that the amount of time they will prioritize to spend on an intervention that, in their view, does not directly support daily operation, is limited. Nielsen [39] suggest that intervention activities should be incorporated into the line managers’ daily work. In relation to the sickness absence intervention, the SA coordinators could consider trying to incorporate the activities into already existing work tasks so that the line managers do not perceive it as another responsibility to take care of in a busy workday. For example, if the activities could be incorporated into already existing meetings between the line manager and the employees rather than extra meetings or use existing tools that the line managers already know, instead of developing new ones.

Finally, the role and challenges of SA coordinators uncovered in this study can also be viewed through the lens of the intervention framework’s other four elements (see the "Context" Section). Unsurprisingly, the other components have helped shape the SA coordinator role For example, the role of coordinators reflects the requirements of the other four components, e.g. the requirement to ensure that managers have skills in managing sickness absence (component 1) and that managers receive sparring on the working environment (component 2) and data (component 3). The challenges mentioned are particular reflected in component 4: establishment of a decision-making forum. This component aims to enhance support and involvement in the intervention, clarify roles and responsibilities regarding sickness absence and set realistic goals for the intervention that are tailored to each unit. The challenges voiced by the SA coordinators suggest that achieving this purpose can be difficult. For example, the findings suggests that the role and responsibilities of those involved in the intervention were not clear, and the SA coordinators lacked organizational support to define and establish their role, which challenged their collaboration with some line managers. Moreover, the perceived lack of line manager engagement in the intervention might be due to the failure to create ownership of the intervention at line manager level.

Furthermore, it is arguable that the organizational (outside) anchoring of the role makes it challenging for the SA coordinators to tailor the intervention to each unit, as it is essential to thorough understand the realities of the units to be able to tailor the intervention to the different units’ different needs.

### Strengths and Limitations

This study gives insight into the challenges that may occur when introducing a new organizational role. It adds to the existing literature on sickness absence management by providing knowledge about some of the challenges a designated SA coordinator may experience in their job. It also adds to implementation research with knowledge about what to consider if designating a person to facilitate implementation processes. The results are useful for workplaces that need to implement similar interventions. In particular, individuals in positions similar to SA coordinators can utilize this knowledge when developing and implementing interventions.

The main strength is the combined use of two qualitative approaches. Our reason for including both interviews and observations was to gain a thorough understanding of the roles and responsibilities of the SA coordinators in practice. Furthermore, supplementing the interviews with observations increases credibility as “what someone tells you in an interview can be checked against what you observe on site” [40].

The limitation of the study relates to transferability. As the SA coordinators examined in this study emerged as part of a new initiative in Denmark, the use of SA coordinators is limited to the workplaces participating in the initiative. The results may therefore only apply for these workplaces. However, as there are some overlaps with the role of RTW coordinators, the findings are likely to be relevant for workplaces using RTW coordinators to facilitate RTW processes, as it is possible that the two roles share the same experiences and challenges.

Another limitation is the small sample size. This study gives insight into the role of four SA coordinators and it is possible that other SA coordinators have other responsibilities and experiences than these four. However, having interviewed each SA coordinator twice at different times did provide us with a throughout understanding of the role, as it gave us the opportunity to explore the role both in real time and retrospect. Also, as most of the SA coordinators work in organizations within health care or day care, results are likely to be representative for this setting, whereas extrapolation to other organizational contexts, such as education should be done with care.

Furthermore, data were collected during the COVID-19 pandemic, and it must be assumed that this has influenced the participants’ experiences of the implementation process. For instance, it can be assumed that the experienced lack of engagement from line managers was more likely due to a focus on adhering to restrictions and reducing the risk of infection rather than a lack of support for the initiatives. It is likely that if data were collected later, the results would be different.

Lastly, this study only examines the role from the perspectives of the SA coordinators. It is necessary to also explore how the line managers perceive the role and how they experience collaborating with them in order to fully understand how the role works in practice.

## Conclusion

The SA coordinators in this study largely shared similar work duties, which primarily related to collaborating with line managers in managing sickness absence. The challenges highlighted in this study include experiences of lack of engagement from the line managers to participate actively in the intervention and that the line managers did not perceive the SA coordinators as a valuable function.

The findings suggest that, in order for the SA coordinators to be able to carry their role efficiently, it seems important to establish commitment to the role among the line managers in order to enhance good working relationships and collaboration.

## Data Availability

The datasets generated and/or analyzed during the current study are not publicly available as they are in Danish and not translated but are available from the corresponding author on reasonable request.
